# Effect of solution-focused approach on anxiety and depression in patients with rheumatoid arthritis: A quasi-experimental study

**DOI:** 10.3389/fpsyg.2022.939586

**Published:** 2022-12-13

**Authors:** Chunli Zhang, Xuehua Wu, Ying Yuan, Huamei Xiao, Erhui Li, Hongyan Ke, Mei Yang, Xiaodong Zhu, Zhicheng Zhang

**Affiliations:** ^1^Department of Nursing, Huanggang Central Hospital of Yangtze University, Huanggang, Hubei, China; ^2^Department of Hand Surgery, Union Hospital, Tongji Medical College, Huazhong University of Science and Technology, Wuhan, China; ^3^Department of Neonatology, Huanggang Central Hospital of Yangtze University, Huanggang, Hubei, China; ^4^Department of Neurology, Huanggang Central Hospital of Yangtze University, Huanggang, Hubei, China; ^5^Department of Endocrinology, Huanggang Central Hospital of Yangtze University, Huanggang, Hubei, China; ^6^Department of Oncology, Huanggang Central Hospital of Yangtze University, Huanggang, Hubei, China; ^7^ Department of Gastroenterology, Tongji Hospital, Tongji Medical College, Huazhong University of Science and Technology, Wuhan, China

**Keywords:** rheumatoid arthritis, solution-focused approach, anxiety, depression, arthritis self-efficacy

## Abstract

**Introduction:**

Anxiety and depression are common psychological problems in rheumatoid arthritis (RA) patients. However, few effective nursing intervention models have been designed specifically to improve anxiety and depression in RA patients. Solution-focused approach (SFA) is an effective intervention method for psychosocial issues. There have been no studies involving SFA yet in RA patients. This study investigated the effects of SFA-based nursing intervention on anxiety and depression in RA patients.

**Methods:**

A quasi-experimental study using a convenience sampling of RA patients was conducted. The 48 RA patients were divided into the control group (*n* = 24) and the experimental group (*n* = 24). The control group received routine nursing intervention, while the experimental group received SFA-based nursing intervention. The scores on the self-rating anxiety scale (SAS), self-rating depression scale (SDS), arthritis self-efficacy scale-8 (ASES-8), and questionnaire on patient satisfaction with nursing care were collected before and after nursing interventions.

**Results:**

*Between-Group Comparison*: Before the nursing intervention, there was no statistically significant difference in the SDS, SAS, and ASES-8 scores between the two groups (*p* > 0.05). However, after the nursing intervention, the SDS and SAS scores of the experimental group were statistically significantly lower than those of the control group (*p* < 0.05). In contrast, the ASES-8 score of the experimental group was statistically significantly higher than that of the control group (*p* < 0.05). In addition, patient satisfaction with nursing care of the experimental group was better than that of the control group (*p* > 0.05). *Within-Group Comparison*: There was no statistically significant difference in the SDS, SAS, and ASES-8 scores in the control group before and after routine nursing intervention (*p* > 0.05). However, in the experimental group, the SDS and SAS scores before SFA-based nursing intervention were statistically significantly higher than those after SFA nursing intervention (*p* < 0.05), and the ASES-8 score before SFA-based nursing intervention was considerably lower than that after SFA nursing intervention (*p* < 0.05).

**Discussion:**

SFA-based nursing intervention can effectively improve anxiety, depression, and arthritis self-efficacy of RA patients. This study broadens clinical psychological nursing intervention models for RA patients. SFA may be an effective nursing model for various psychosocial problems in the current medical context.

## Introduction

### Rheumatoid arthritis

Rheumatoid arthritis (RA) is a chronic inflammatory condition that mainly affects the joints (Gautam et al., 2020). RA has a variety of systemic manifestations, including arthritis pain, fatigue, morning stiffness, anemia, and weight loss (Favalli, 2020). Some common comorbidities include osteoporosis, cardiovascular disease, diabetes, infection, malignancies, depression, sleep disturbances, and other mental disorders (Tournadre et al., 2019). The prevalence of RA is 0.5–1% in European and North-American populations. The RA incidence in women is three times higher than in men, but this ratio decreases with age at onset (Intriago et al., 2019). The demography of RA in the western world is changing. More than 50% of RA patients are over 65 years old at diagnosis (Eriksson et al., 2013). The etiology of RA is still unclear. RA medications (non-steroidal anti-inflammatory drugs, corticosteroids, disease-modifiers, and biologic agents) are not curative (Lin et al., 2020). The long-term treatment and adverse reactions may lead to many psychological problems, which reduce treatment compliance, curative effects, and quality of life in RA patients (Berner et al., 2018).

### Incidence of anxiety and depression in RA patients

Anxiety and depression are the most common emotional problems found in RA patients. The incidence rate is much higher than that of the general population and is inconsistent in different areas (Hitchon et al., 2020). Isik et al. reported that the total prevalence of anxiety, depression, and mixed anxiety-depressive disorder was 70.8% in the RA patient group and 7.3% in the control group of the general population (Isik et al., 2007). Of RA patients, 41.5% were found to have depression, 13.4% anxiety, and 15.9% mixed anxiety-depressive disorder (Isik et al., 2007). Hitchon et al. found that the prevalence of current depression in RA patients was 11.3%, generalized anxiety disorder was 7.3%, and any anxiety disorder was 19.3% (Hitchon et al., 2020). Katchamart reported that 12.5 and 14.5% of RA patients had some degree of depression and anxiety, respectively, in the Siriraj Rheumatoid Arthritis Registry or the Thai Army Rheumatoid Arthritis Cohort (Katchamart et al., 2020). Kwiatkowska et al. also confirmed that the incidence of depression in RA patients is two to three times that of individuals without RA (Kwiatkowska et al., 2019). Longitudinal studies suggest cumulative risk for depression and intermittent recurrence over time (Wolfe and Michaud, 2009).

Anxiety and depression are often difficult to identify in RA patients. They are generally reluctant to discuss their anxiety and depression with family members and medical personnel because they fear it will harm their image (Machin et al., 2020). Some patients do not even carry out relevant examinations and consider negative emotions to be normal responses to chronic diseases. Furthermore, diagnosing depression in patients with RA is a complicated process. There is an overlap in the symptoms of depression and RA (for example, fatigue, weight loss, insomnia, and lack of appetite), so the depression frequently goes unrecognized.

### Reasons for anxiety and depression in RA patients

It is necessary to fully understand the possible reasons for anxiety and depression in RA patients. Some reasons include joint deformities, impaired joint function, inability to work, and personal economic losses (Fiest et al., 2017). Depression is associated with increased pain (Vergne-Salle et al., 2020), reduced health-related quality of life (Zhang et al., 2020), increased levels of physical disability (Carpenter et al., 2020), and increased health care costs in RA patients (Joyce et al., 2009). Severe pain, frequent treatment, and low income would also aggravate the state of anxiety and depression in RA patients (Espinoza et al., 2021). The association between pain and RA depression has remained statistically significant even after the degree of disease activity has been controlled (Margaretten et al., 2011). It is often not difficult to identify depression and anxiety secondary to rheumatoid arthritis, and the extent of depression and anxiety is often positively related to the disease condition.

### Adverse outcomes of anxiety and depression in RA patients

Anxiety and depression in RA patients can lead to undesirable consequences, including increased pathological activity, poor tolerance for joint pain, decreased physical function, and low adherence to therapy (Machin et al., 2020). RA impacts work capacity in the Indian population. Manual jobs and the absence of medical insurance predict leaving the labor force before the official retirement age (Alleva et al., 2018). RA patients with depression had a greater relative risk of utilization of emergency services, RA-related hospitalizations, days spent in the hospital, and RA-related surgeries than RA patients without depression (Li et al., 2019). RA patients with depression had more days of short-term disability than patients without depression (Li et al., 2019). Anxiety and depression can cause sleep disturbances in RA patients (Silva et al., 2020). In RA patients, depression also interacts with the way patients cope with their physical illness and how they interact with their rheumatologist (Dickens and Creed, 2001). The simultaneous presence of anxiety and depression may lead to suicide and aggravate the prognosis of RA patients (Beşirli et al., 2020).

### Solution-focused approach

Nursing intervention is a critical approach to managing negative emotions. However, few effective nursing intervention models have been designed specifically to improve anxiety and depression in RA patients (Machin et al., 2017). Most interventions have been based on RA patients’ education. Educational interventions have had limited effectiveness in changing behavior (Song et al., 2020). Therefore, it is necessary to develop evidence-based, pragmatic, patient-centered interventions to help reduce negative emotions in RA patients.

Solution-focused approach is initially established as a new way of counseling by Steve de Shazer, Insoo Kim-Berg, and other colleagues in the 1980s (de Shazer and Berg, 1997). SFA is a strengths-based, resource-based, and goal-directed therapy model. SFA guides individuals to set goals and solve problems with exceptionally positive experiences. SFA can fully explore the potential of individuals by driving individuals to formulate scientific and feasible plans. SFA can also stimulate their initiative and enhance their ability to manage themselves correctly to solve the current problems.

It has been demonstrated that SFA is effective in the psychological intervention of many nursing jobs, such as psychological distress of young adolescent patients with cancer (Zhang et al., 2021), the mental health of pregnant women (Ramezani et al., 2017), and intimacy of children with disabilities (Baldwin et al., 2013). Generally, SFA includes five key steps: ① describing problems; ② constructing goals; ③ exploring exceptions; ④ giving feedback; and ⑤ evaluating progress. SFA can effectively mobilize enthusiasm, improve self-management efficiency, and reduce negative emotions in individuals (Ma et al., 2021).

There are few effective nursing models for RA patients with anxiety and depression. SFA is an effective intervention method for psychosocial issues in the current medical environment (Zhang et al., 2021). Therefore, we hope to explore new and more effective nursing models to help address the psychological problems of RA patients. However, SFA has not yet been applied to RA. Hence, the study aims to explore the effects of SFA-based nursing intervention on RA patients’ anxiety, depression, and self-efficacy.

## Materials and methods

### Participants and groupings

This study was a quasi-experimental study using a convenience sampling of RA patients. The inclusion criteria include as follows: (1) Meeting the 2010 ACR/EULAR criteria for RA (Kay and Upchurch, 2012); (2) Ages between 18 and 75 years old; (3) Self-rating anxiety scale (SAS) score ≥ 50 points and Self-rating depression scale (SDS) score ≥ 53 points. The exclusion criteria: (1) RA patients were combined with other chronic serious diseases, such as heart, brain, and kidney diseases; (2) The hospital stay was less than 7 days. The 48 RA patients were divided into the control group (*n* = 24) and the experimental group (*n* = 24) enrolled from February 2019 to July 2019. The control group received routine nursing intervention, while the experimental group received SFA-based nursing intervention.

### Nursing interventions

#### The control group

Routine nursing interventions for RA patients were performed. The main details include as follows: ① Keep bed rest during acute stages; ② Attention should be paid to keeping warm, moisture-proof, and cold-proof; ③ Carry out routine health education about daily joint function exercises; ④ Strengthen dietary nutrition; ⑤ Calcium supplementation, and more sun exposure; ⑥ Guide patients to take medicine; and ⑦ Pay attention to limb activity and psychological changes.

#### The experimental group

Solution-focused approach-based nursing intervention was performed by the nursing team consisting of four nurses, one psychologist, and one attending physician. The experimental group received four times of SFA-based nursing interventions on the second day after admission, 1 week after admission, discharge day, and 2 weeks after discharge. The duration of each intervention was around 30–60 min. Intervention forms include: ① During hospitalization: face-to-face communication in the ward; and ② After discharge: follow-up by phone, WeChat, and door-to-door follow-up. Researchers should discuss with patients about follow-up methods in advance before discharge. Telephone communication intervention is the first choice; WeChat and door-to-door follow-up methods are optional.

### Steps of SFA-based nursing intervention

① **Describe problems:** The patient’s current issues with their impact on the patient’s quality of life should be clarified. For example, when a patient has recently had frequent joint pain or morning stiffness, the researcher can ask: “How long has your joint pain or morning stiffness been, how severe it is, and whether it seriously affects your quality of life?”
② **Construct goals:** The patient should be encouraged that their future life quality will be improved if the current problem is solved. Researchers try to make patients feel good in advance, assuming the problem is solved. Then researchers discuss with patients how to establish practical goals and specific solutions. For example, “Assuming that your morning stiffness and joint pain are already healed, will you feel that life is much better than now?” or “To what extent do you hope to improve?”
③ **Explore exceptions:** The exceptional cases and experiences in which the above problems have been perfectly resolved in patients’ lives are explored through communications. Under these circumstances, the patient’s efforts to resolve current issues should be used as a reference in the subsequent recovery. For example, “Did you have joint pain, or when did morning stiffness improves; if so, what efforts did you make under those conditions?” If there were no exceptionally positive experiences, researchers would discuss with patients and construct new solutions.
④ **Positive feedback:** The problems, goals, and related exceptions are positively summarized in the above three steps to establish feasible solutions and enhance the patient’s self-confidence. For example, “Your main problems are joint pain and morning stiffness. You want to be fully relieved from the disease and have a better life. You can then refer to the previous exception as the solution.” At this stage, scaled questions can be used to build more specific and clear goals. For example, “If you use 0–10 points to represent your morning stiffness, 0 points to represent the most severe morning stiffness you have experienced, and 10 points to represent the lightest morning stiffness, you can rate how many points you hope to achieve in the future.” If the goals were not achieved ideally, researchers would adjust the SFA-based nursing intervention plan. If the constructed goals were not achieved well, researchers would then get back to the third step to explore exceptionally positive experiences.
⑤ **Evaluate progress:** The goals achieved by the patient through previous efforts should be positively evaluated, enhancing the patient’s self-confidence to solve their problems gradually. For example, “You have done a good job so far, and you have made great progress in improving your morning stiffness score. Congratulations on your fantastic work!” If patients had achieved their goals, researchers would then go back to the second step to construct new higher goals until patients achieved satisfactory outcomes.

**Figure 1:**
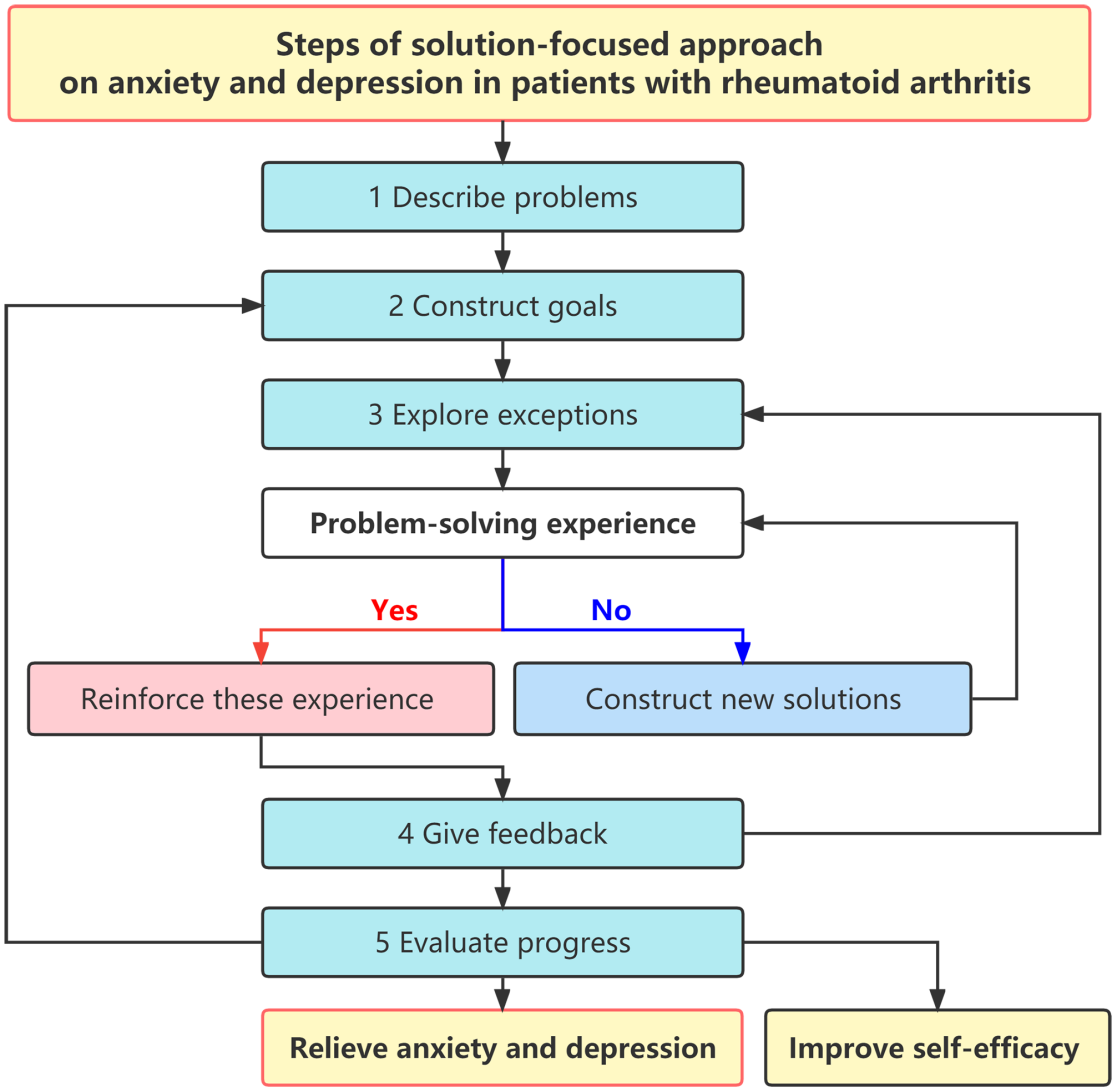
Steps of solution-focused approach on anxiety and depression in patients with rheumatoid arthritis.

All the above steps of SFA-based nursing on anxiety and depression in RA patients were summarized in Figure 1.

### Evaluation tools

#### Self-rating anxiety scale

The anxiety self-rating scale (SAS) was compiled by William W.K. Zung in 1971 (Zung, 1971) and is mainly used to assess the anxiety degree of patients. There are 20 questions, of which 15 questions are forward scoring, and five questions are reverse scoring (items 5, 9, 13, 17, and 19). Each question has four scoring levels: ① No or very little time; ② a small part of the time; ③ a considerable amount of time; ④ most or all of the time. For positive scoring questions, ①, ②, ③, and ④ mean 1, 2, 3, and 4 points, respectively. For reverse scoring questions, ①, ②, ③, and ④ mean 4, 3, 2, and 1 points, respectively. The total score of the 20 items will be calculated and then multiplied by the coefficient of 1.25. The SAS scores can be further divided into three levels: mild anxiety: 50–59 points; moderate anxiety: 60–69 points; and severe anxiety: >69 points. Cronbach’s coefficient of SAS is 0.875 (Zung, 1971).

#### Self-rating depression scale

The self-rating depression scale (SDS) was compiled by William W.K. Zung in 1967 (Zung, 1967). It is mainly used to assess the depression degree of patients. Ten questions are forward scoring, and 10 questions are reverse scoring. The 20 questions can be divided into four categories: two questions for psycho-emotional symptoms, eight questions for somatic disorders, two questions for psychomotor disorders, and eight questions for depressive psychological disorders. Each question has four scoring levels: ① none or very little time; ② a small part of the time; ③ a considerable amount of time; and ④ most or all of the time. Each question has four scoring levels: ① No or very little time; ② a small part of the time; ③ a considerable amount of time; and ④ most or all of the time. For positive scoring questions, ①, ②, ③, and ④ mean 1, 2, 3, and 4 points, respectively. For reverse scoring questions, ①, ②, ③, and ④ mean 4, 3, 2, and 1 points, respectively. The total score of the 20 items will be calculated and then multiplied by the coefficient of 1.25. The SDS scores can be further divided into three levels: mild depression: 53–62 points; moderate depression: 63–72 points; and severe depression: >72 points. Cronbach’s coefficient of SAS is 0.796 (Zung, 1967).

#### Arthritis self-efficacy scale-8

The arthritis self-efficacy scale-8 (ASES-8), including eight items, was developed based on the arthritis self-efficacy scale by Lorig in 1989 (Lorig et al., 1989). ASES-8 was used to evaluate the self-efficacy of RA patients. Scoring on each item ranges from 1 to 10, meaning “very un-confident” to “very confident.” The overall score is the average of all eight items. The higher the score, the higher the patient’s sense of self-efficacy. Cronbach’s coefficient of ASES-8 is 0.0.942 (Lorig et al., 1989).

#### Questionnaire on patient satisfaction with nursing care

The questionnaire on patient satisfaction with nursing care at our hospital adopted a 100-point scale. Very satisfied: total score ≥ 80 points; satisfied: total score 60–79 points; dissatisfied: total score < 60 points. Calculation of patient satisfaction with nursing care: (very satisfied + satisfied) patients/the total number of patients. Cronbach’s coefficient is 0.873.

### Data collection

**Figure 2:**
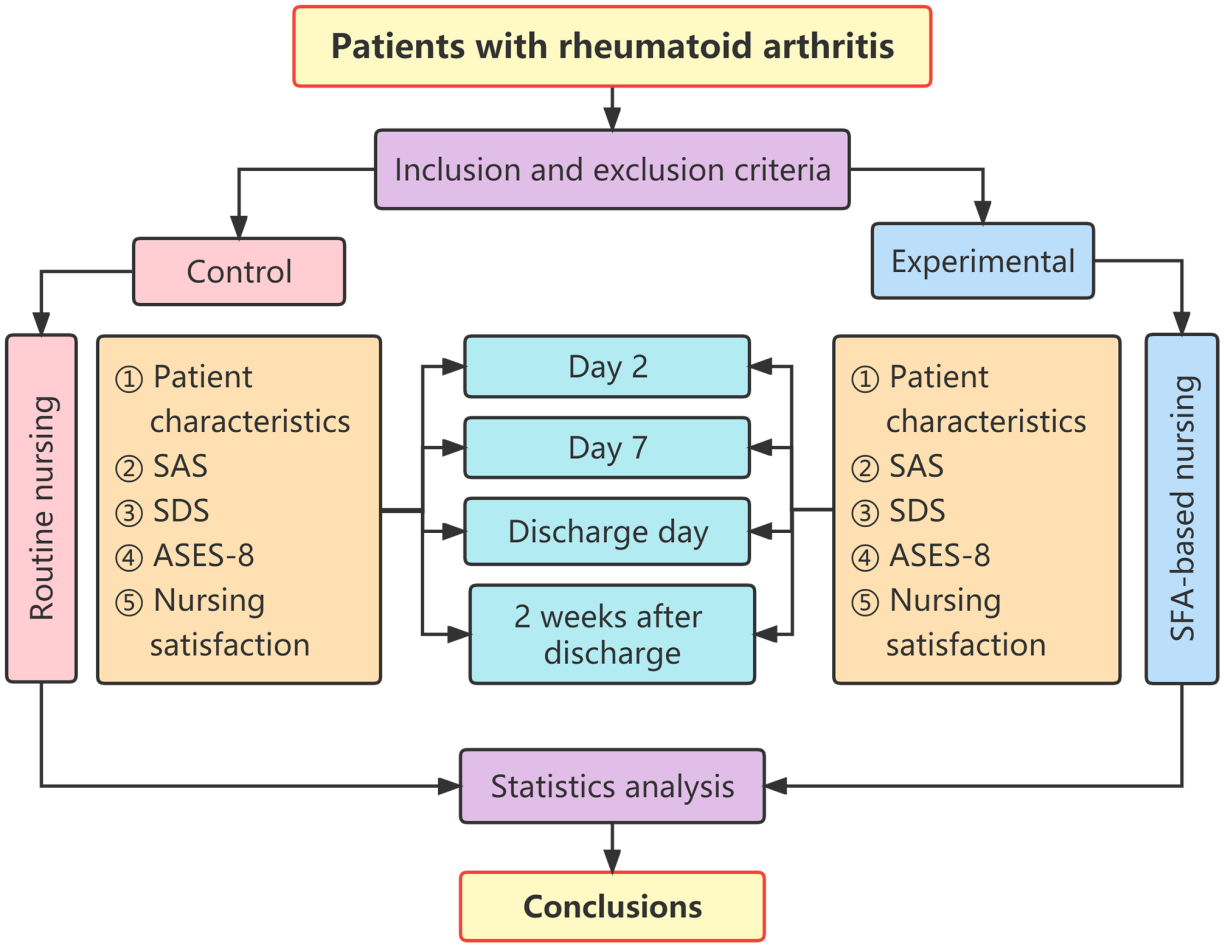
The timepoints and content of evaluation.

The responsible investigator collected all relevant clinical data and questionnaire records for each patient. The purpose of the study was explained to patients. The patient signed the informed consent. When the patient filled in the questionnaire, if there was any doubt, the researcher gave an objective explanation until the patient understood it clearly. If the patient’s cultural skills were poor, the researcher filled in the questionnaire. Each patient completed the questionnaire independently. The questionnaires were collected on the spot and carefully checked. The time points and content of estimation were summarized in Figure 2: ① The patient characteristics questionnaire was conducted on the second day of admission. ②The SAS score, SDS score, ASES-8, and questionnaire on patient satisfaction with nursing care were carried out on the second day after admission, on the seventh day after admission, on discharge, and 2 weeks after discharge.

### Data analysis

All data from the research subjects, including the patient characteristics questionnaire, SAS score, SDS score, ASES-8, and questionnaire on patient satisfaction with nursing care, were analyzed by Graphpad Prism 9.0.0. The λ^2^ test and the Mann–Whitney U test were used to compare the patient characteristics. Paired *t*-tests and two independent sample *t*-tests were used to compare the scores of the two groups. *p* < 0.05 indicates statistical differences.

## Results

### Between-group comparison of patient characteristics

Table 1 presents the patients’ demographics and clinical characteristics. The demographic characteristics, such as age, gender, educational level, average monthly income, residence, marital status, social support, payment method, and occupation of the two groups, were analyzed. The majority of patients are female with ages greater than 40 years old and low monthly income. There were no statistically significant differences in demographic characteristics between the control and experimental groups (*p* > 0.05). Moreover, no statistically significant difference was found in the RA condition characteristics, such as disease duration, morning stiffness time, or joint function. Therefore, the two groups of patients were comparable.

**Table 1:** Between-group comparison of patient characteristics.

Factors		Control	Experimental	*λ^2^/U*	*p*
**Age (years)**	20 ~ 40	9	11	0.342	0.558
	≥ 41	15	13		
**Gender**	Male	8	9	0.091	0.762
	Female	16	15		
**Educational level**	≤ High school	13	16	0.784	0.376
	≥ College	11	8		
**Monthly income (yuan)**	≤ 5,000	16	15	0.091	0.763
	> 5,000	8	9		
**Residence**	Rural	11	12	0.083	0.773
	Town	13	12		
**Marital status**	Married	18	19	0.117	0.731
	Other	6	5		
**Social support**	Living alone	6	4	0.505	0.477
	Home	18	20		
**Payment methods**	At own expense	4	5	0.136	0.712
	Medical insurance	20	19		
**Profession**	On-the-job	18	19	0.118	0.731
	Other	6	5		
**Disease duration**	1–10 years	13	14	0.085	0.771
	>10 years	11	10		
**Morning stiffness time**	≤15 min	10	11	0.367	0.713
	16–59 min	9	9		
	≥ 60 min	5	4		
**Joint function**	Class I	4	3	0.143	0.886
	Class II	10	12		
	Class III	7	7		
	Level IV	3	2		

### Between-group comparison of SAS, SDS, and ASES-8 before and after nursing intervention

Before the nursing intervention, there was no statistically significant difference in the SAS scores between the experimental group (55.633 ± 1.188) and the control group (56.136 ± 1.036; *p* > 0.05). The SDS scores between the experimental group (59.461 ± 0.940) and the control group (59.255 ± 0.969; *p* > 0.05) showed no statistically significant difference. There was no statistically significant difference in the ASES-8 scores between the experimental group (6.463 ± 0.108) and the control group (6.425 ± 0.179; *p* > 0.05; Table 2).

**Table 2:** Between-group comparison of SAS, SDS, and ASES-8 scores before and after nursing interventions (points, ^**−**^*x* **±** *s*).

	**Items**	**Control**	**Experimental**	***t***	***p***
**Before intervention**	**SAS**	56.136 ± 1.036	55.633 ± 1.188	1.566	0.124
	**SDS**	59.255 ± 0.969	59.461 ± 0.940	0.748	0.459
	**ASES-8**	6.425 ± 0.179	6.463 ± 0.108	0.891	0.378
**After intervention**	**SAS**	55.586 ± 1.060	47.081 ± 1.339	24.4	<0.001
	**SDS**	58.815 ± 1.014	49.922 ± 0.981	30.88	<0.001
	**ASES-8**	6.488 ± 0.182	7.267 ± 0.090	18.8	<0.001

After the last nursing intervention, the SAS score of the experimental group (47.081 ± 1.339) was statistically significantly lower than that of the control group (55.586 ± 1.060; *p* < 0.05); The experimental group (49.922 ± 0.981) has statistically significantly lower SDS score than the control group (58.815 ± 1.014; *p* < 0.05). The SDS score of the experimental group (7.267 ± 0.090) was statistically significantly higher than that of the control group (6.488 ± 0.182; *p* < 0.05; Table 2).

### Within-group comparison of SAS, SDS, and ASES-8 scores before and after intervention

In the control group, there was no statistically significant difference in the SAS score before routine nursing intervention (56.136 ± 1.036) and after routine nursing intervention (55.586 ± 1.060; *p* > 0.05). The SDS scores before routine nursing intervention (59.256 ± 0.969; the first time) and after routine nursing intervention (58.815 ± 1.014; the last time) showed no statistically significant difference (*p* > 0.05). In the control group, there was no statistically significant difference in the ASES-8 score before routine nursing intervention (6.425 ± 0.179; the first time) and after routine nursing intervention (6.488 ± 0.182; the last time; *p* > 0.05; Table 3).

**Table 3:** Within-group comparison of SAS, SDS, and ASES-8 scores before and after nursing intervention (points, ^**−**^*x* ± *s*).

	**Items**	**Before intervention**	**After intervention**	***t***	***p***
**Control**	**SAS**	56.136 ± 1.036	55.587 ± 1.060	1.815	0.076
	**SDS**	59.255 ± 0.969	58.815 ± 1.014	1.54	0.13
	**ASES-8**	6.425 ± 0.179	6.488 ± 0.182	1.209	0.232
**Experimental**	**SAS**	55.633 ± 1.188	47.083 ± 1.339	23.4	<0.001
	**SDS**	59.461 ± 0.940	49.922 ± 0.981	34.4	<0.001
	**ASES-8**	6.463 ± 0.108	7.267 ± 0.090	28.02	<0.001

In the experimental group, the SAS score before SFA-based nursing intervention (55.633 ± 1.188; the first time) was statistically significantly higher than that after SFA-based nursing intervention (47.081 ± 1.339; the last time; *p* < 0.05). The SDS score before SFA-based nursing intervention (59.461 ± 0.940; the first time) was statistically significantly higher than that after SFA-based nursing intervention (49.922 ± 0.981; the last time; *p* < 0.05). The ASES-8 score before SFA-based nursing intervention (6.463 ± 0.108; the first time) was statistically significantly lower than that after SFA-based nursing intervention (7.267 ± 0.090; the last time; *p* < 0.05; Table 3).

### Between-group comparison of patient satisfaction with nursing care

Patient satisfaction with nursing care of the experimental group (96%) was better than that (92%) of the control group. However, there was no statistically significant difference between them (*p* > 0.05; Table 4).

**Table 4:** Between-group comparison of patient satisfaction with nursing care(%).

**Groups**	**Very satisfied**	**Satisfied**	**Dissatisfied**	**Satisfaction (%)**	***U***	***p***
**Control**	7	14	3	92	1.396	0.163
**Experimental**	12	10	2	96		

## Discussion

Rheumatoid arthritis patients often experience anxiety and depression. The average SAS and SDS scores of all participants in our study were less than 60, indicating that anxiety and depression in RA patients were mainly mild. The results showed that SFA-based nursing intervention could statistically significantly improve the anxiety, depression, and arthritis self-efficacy of RA patients in the experimental group. In contrast, routine nursing intervention could not effectively improve those indicators of RA patients in the control group.

### SFA could improve negative emotions

Solution-focused approach has a more significant advantage than the traditional psychological nursing model regarding patient psychological intervention (Wright et al., 2014). It fully mobilizes the patient’s enthusiasm so that the patient can be proactive and confident in solving their problem. SFA has been demonstrated to be an effective intervention in mental disorders and clinical nursing research as follows.

#### The application of SFA in mental disorder researches

Solution-focused approach has been widely used in many settings, including family services, mental health, child care, public health services, and psychotherapy centers. SFA could address Autism Spectrum Disorder (ASD)-related concerns within the family and be generalizable to reduce the additional stress of care coordination between parents and various ASD specialists (Parker et al., 2020). Nurses in the adolescent mental health field can use SFA to improve self-efficacy and self-esteem in adolescents with attention-deficit/hyperactivity disorder (Karakaya and Özgür, 2019). Socially withdrawn children can benefit from a group SFA intervention and reach their goals, probably through sharing their feelings, experiences, and support. SFA may be suitable for school nurses working with children with special needs. SFA groups are a recommended measure for use in school health services (Kvarme et al., 2010). SFA can give an occupational healthcare staff valuable tools to positively influence their relationships with patients (Mishima et al., 2005). Lee et al. evaluated SFA effects on domestic violence offenders and found a statistically significant increase in their self-esteem based on self-reports (King and Batagol, 2010). Schott et al. found that SFA complements the principles of psychiatric rehabilitation and is a recovery intervention for empowering persons with severe mental illness (Schott and Conyers, 2003).

#### The application of SFA in clinical nursing research

There have been several clinical nursing studies with SFA applications recently. SFA resulted in a statistically significant reduction in the psychological distress and improvement in the hope of adolescent and young adult patients with cancer (Zhang et al., 2021). Ramezani et al. found that the integration of SFA and cognitive-behavioral counseling programs in prenatal care can effectively improve the mental health of pregnant women (Ramezani et al., 2017). Short-term SFA interview technique intervention may affect overweight and obese individuals’ nutrition and exercise behaviors. This intervention can reduce the risk of obesity-related diseases, minimizing repeated hospital admissions (Akgul Gundogdu et al., 2018). Nurses displayed moderate anxiety, and SFA thinking skills enable them to quickly organize and manage care processes in extraordinary circumstances such as pandemics (Selçuk Tosun et al., 2021). SFA could offer a promising method for implementing a strengths-based, relational, and goal-oriented intervention approach to working with families and children with disabilities (Baldwin et al., 2013). SFA may be a helpful approach to the training of communication skills. It provides a structured and easily understood toolkit that is harmonious with nursing values of empowerment (Bowles et al., 2001).

#### SFA could improve anxiety and depression in RA patients in this study

This study confirms the effect and value of SFA in improving anxiety and depression in RA patients. Compared with routine nursing, the SFA-based intervention could better stimulate patients’ self-confidence and lay a foundation for the long-term solution of psychological problems. Our results are consistent with the above research conclusions.

### SFA could improve self-efficacy in RA patients

Self-efficacy is a psychosocial variable that has been defined as the individual’s confidence to perform a specific task. Self-efficacy is considered the central motor of developing human motivation, psychosocial well-being, and personal achievement. Higher levels of SE are associated with more willingness to take risks and a sense of accomplishment (Picha and Howell, 2018).

Self-efficacy seems essential in managing RA. Unpredictable courses of RA could make patients feel their condition is uncontrollable and decrease their self-efficacy in handling it (Carrick and Randle-Phillips, 2018). Self-efficacy contributes to self-management behavior and promotes psychological adjustment to chronic illness (Yang et al., 2021). There is an association between higher self-efficacy and greater goal achievement, positive affect, acceptance of the disease, problem-solving coping, physical function, physical activity participation, and quality of life in RA patients (Hosseini Moghadam et al., 2018). A recent systematic review of the role of self-efficacy in patients with RA similarly noted an association between high self-efficacy and positive affect, physical function, and ability to participate in social roles and activities (de Ridder et al., 2008). Therefore, improving the self-efficacy of RA patients is necessary.

Several studies have shown that SFA has the effect of increasing self-efficacy. Midwifery care based on SFA provided by online synchronous video conferencing during the COVID-19 pandemic is an effective and safe method to reduce the fear of childbirth in women and increase their self-efficacy (Kaya and Guler, 2022). Learning and developing communication skills fundamental to SFA thinking increases nursing students’ confidence in individuals and improves their self-efficacy(Akgül-Gündoğdu and Selçuk-Tosun, 2021). SFA nursing can alleviate leukemia chemotherapy patients’ negative emotions and cancer-related fatigue, improve their coping styles, and increase their self-efficacy and quality of life (Wang et al., 2021). Simm et al. introduced that SFA helps the clinician tap into patient expertise and develop detailed descriptions of the patient’s preferred future, enhancing self-efficacy and empowerment (Simm et al., 2014). This study also found that SFA-based nursing intervention could improve the self-efficacy of RA patients.

### SFA enhances patient satisfaction with nursing care in RA patients in this study

Solution-focused approach-based nursing intervention could enhance the communication and relationships between patients and nurses. SFA is a feasible and effective method for nursing advanced schistosomiasis patients. It improves patient satisfaction with nursing care and the trustiness of the patients with the health care providers (Hong-Mei et al., 2016). This study also found that SFA could improve patient satisfaction with nursing care in RA patients.

### Limitations

It is worth stressing a few limitations of the study. First, this is a single-center clinical trial, limiting the generalizability of the findings. The application of SFA in clinical practice needs to be more widely used, and the SFA should be gradually improved through more practice. Second, the sample size used in this study is relatively small, limiting a more reliable analysis and conclusion of SFA. Future research should consider such investigation when a larger sample size becomes available. Third, the follow-up time of the patients in this study was relatively short without longer-term follow-up due to infeasibility and limited resources. Future research should consider 3-, 6-, and 12-month follow-up time points to clarify the long-term effect of SFA further.

In this study, we measured each measure separately at four different time points, which led to the seemingly feasible possibility of a multi-group ANOVA analysis. However, we only used *t*-tests instead of ANOVA analysis for the following reasons. The changes in the second and third estimation scores serve as the adjustment basis for the individualized SFA intervention plan, but do not serve as the final indicator for evaluating the SFA effect. We only compared two sets of data at a time: the first measurement (before the intervention) and the fourth measurement (the last, after the intervention), which allows complete estimation of the overall SFA effect. Therefore, no ANOVA analysis was performed.

There are a few side effects during implementing SFA. First, nurses must accept the goals set by patients themselves, even if they think they are unrealistic. Second, it is always the patient, not the nurse, that is praised, and this may detract from the nurse’s motivation when treating the patient. Third, helping others is in the nature of many people. However, SFA primarily emphasizes that patients themselves seek solutions to current problems, and nurses must resist the urge to offer their own “good ideas.”

Some limitations of self-administered assessments exist in assessing anxiety and depression when using our SAS and SDS scales. ① Honesty: Patients may be more inclined to provide socially acceptable answers than they actually are, thereby lowering their anxiety and depression scores. ② Introspective ability: Patients may not be able to accurately assess their anxiety and depression, and are prone to exaggeration or reduction of their emotional state. ③ Numerical scales: Our self-rating anxiety and depression scales are numerical and may cause patients to give extreme or moderate assessments of various issues.

## Conclusion

The combined effects of RA with anxiety and depression will seriously affect the life quality of RA patients. SFA-based nursing could statistically significantly improve anxiety, depression, and arthritis self-efficacy. This study broadens clinical psychological nursing intervention models for RA patients. Further, SFA may be an appropriate clinical approach for nursing professionals in various clinical settings to monitor health-related behaviors and support effective care coordination that transcends disciplinary distinctions.

## Data availability statement

The original contributions presented in the study are included in the article/supplementary material, further inquiries can be directed to the corresponding authors.

## Ethics statement

The studies involving human participants were reviewed and approved by Huanggang Central Hospital of Yangtze University. The patients/participants provided their written informed consent to participate in this study.

## Author contributions

CZ and XW contributed equally to the research design, statistical analysis, and writing of the manuscript. YY, HX, EL, HK, and MY contributed to communication with patients and collection of questionnaires. XZ and ZZ contributed to the project administration and supervision. All authors contributed to the article and approved the submitted version.

## Funding

The research was funded by the Huanggang Central Hospital of Yangtze University, Huanggang, Hubei, China.

## Conflict of interest

The authors declare that the research was conducted in the absence of any commercial or financial relationships that could be construed as a potential conflict of interest.

## Publisher’s note

All claims expressed in this article are solely those of the authors and do not necessarily represent those of their affiliated organizations, or those of the publisher, the editors and the reviewers. Any product that may be evaluated in this article, or claim that may be made by its manufacturer, is not guaranteed or endorsed by the publisher.
